# Sex on the Beach: Sexual Size Dimorphism Among Aquatic Bugs (Nepomorpha) Inhabiting Different Habitats

**DOI:** 10.1002/ece3.71501

**Published:** 2025-06-05

**Authors:** Gábor Bakonyi, Anikó Seres

**Affiliations:** ^1^ Department of Zoology and Ecology Institute for Wildlife Management and Nature Conservation, Hungarian University of Agriculture and Lif Sex on the Beach: Sexual Size Dimorphism Among Aquatic Bugs (Nepomorpha) Inhabiting Different Habitats e Sciences Gödöllő Hungary

**Keywords:** fecundity selection, female‐biased taxa, natural selection, Rensch's rule, sexual dimorphism index, sexual selection

## Abstract

Aquatic bugs (Nepomorpha) are a species‐rich taxon of insects inhabiting all types of freshwaters, often at high densities, and some are among the dominant predators in aquatic food webs. In contrast to the vertebrates and some other arthropod taxa, sexual dimorphism in aquatic bugs is a poorly investigated issue; the present work is the first comprehensive analysis in this field. Morphological differences between the sexes are generally minor, except that female body size is usually longer than that of males. There is no information on the extent of sexual dimorphism in Nepomorpha, except in Belostomatidae and Corixidae. We examined the patterns of sexual size dimorphism (SSD) of 1195 species and subspecific taxa (about 45% of the known species) and whether they conform to Rensch's rule (RR) at the infraorder, superfamily, family and genus levels. We attempted to identify the potential causes of the observed patterns. Our assumption that females are longer‐bodied in most aquatic bug species is falsified. Species living in habitats near the edges of water bodies had higher SSD than in all other habitats. The most likely reason for the large intersexual size difference here is that, unlike the species living in other habitats, the species here are sit‐and‐wait predators. Our results indicate that sexual selection (including fertility selection) plays an important role in the development of SSD. Moreover, it is probable that water‐edge habitats and the associated sit‐and‐wait lifestyle increase sexual selection pressure and the degree of SSD in Nepomorpha. Converse RR, typical for many insects, characterises Nepomorpha as a whole, and two of its superfamilies. At the family and genus levels, however, roughly half of the taxa obey RR and half of them obey converse RR.

## Graphical abstract

We examined the degree of sexual size dimorphism (SSD), based on body length, in approximately 45% of known water bug (Nepomorpha) species. Contrary to our initial hypothesis, we found that females are not significantly longer than males in most species. Our research revealed that habitat plays a significant role in SSD. Species found in nearshore habitats exhibit a higher degree of SSD compared to those in other water bodies. This greater SSD may be associated with a sit‐and‐wait lifestyle.

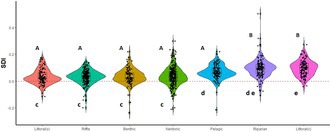

## 1 Introduction

Body size affects an animal's ability to obtain resources, reproduce and survive; thus, it is often subjected to natural selection. Selection is often sex‐specific and may act in different ways on the body length of both male versus female insects (Chown and Gaston 2010). In aquatic bugs, little is known about the extent to which selection effects on males and females are different, or how differences in body size between the sexes affect selection outcomes. Candolin (2004) found that *Sigara falleni* females preferred larger males with larger pala (the pala is a modified, spoon‐like terminal segment of foreleg tarsus of most corixids, used by males, i.a., to hold females during mating). Competition between males, however, favoured the larger individuals with the smaller pala. Consequently, male–male competition and female mate choice were acting oppositely. The importance of male competition in the choice of females depended on environmental factors, in particular the food supply during juvenile development (Candolin 2005). The availability of resources and environmental conditions can vary, leading to differences in the optimal body size for individuals. For four Notonecta species, Svensson et al. (2000) assumed that intraspecific competition is strong because the sexes have similar body sizes. In populations where males and females compete for similar resources, sexual conflict will develop, resulting in sexual size dimorphism (SSD). In this case, however, sexual conflict, if present at all, apparently did not yield SSD. Šigutová et al. (2022) found no difference in habitat preference between males and females of *Notonecta glauca* and *N. obliqua* , despite the sexes of these species not differing in body length (Svensson et al. 2000).

SSD is widely observed in insects. Females are typically larger than males (Teder and Tammaru 2005), which is explained by differences in the reproductive strategies of the sexes. Selection is directed towards the larger body size of females because the larger female body is advantageous for energy accumulation and utilisation, as the larger body size can store more nutrients for egg maturation and deposition. The larger size will enable them to produce more eggs, which will increase their reproductive success (Darwin 1872; Trivers 1972; Pincheira‐Donoso and Hunt 2017). In predatory species, larger females are more successful hunters, giving them more energy for egg production (Blanckenhorn 2005). However, there are insect taxa where males are larger than females (Blanckenhorn et al. 2007; Stillwell et al. 2010). In cases where males compete for females, as in some species of beetles (e.g., Kojima 2019) and crickets (e.g., Baur et al. 2020), males have an advantage in intrasexual competition. In these species, the physical confrontations between males result in larger body sizes which increase the chances of successful fights (Bonduriansky 2001). In insects, SSD can be reflected not only in differences in adult physiology, ecology and behaviour but also in differences in larval growth rates, which may differ between the sexes, resulting in different adult sizes (Teder et al. 2021). Maternal body size and condition may also play a role in determining the sex of offspring, which may contribute to dimorphism (Lynch et al. 2018). Different feeding habits or differences in energy storage of larvae between the sexes also affect the final size of insects (Esperk et al. 2007; Chown and Gaston 2010).

The current number of known aquatic bug (Nepomorpha) species is around 2400 (Wang et al. 2020; Sites 2022). They are distributed worldwide, except Antarctica and the open seas (except Halobates) and occur in a variety of habitats, such as riparian, littoral and benthic habitats, open water and riffle‐pool sequence (Polhemus 2009; Schuh and Weirauch 2020). The taxon is diverse in terms of body structure, shape and size. The smallest species belonging to genera, e.g., *Paraplea*, *Micronecta* and *Tenagobia* are only a few mm in length. The largest species occur in the genus *Lethocerus*. Their body length can reach up to 120 mm (Polhemus and Polhemus 2008). Thus, the differences in body length between the smallest and largest species can reach two orders of magnitude. Like in many other insect taxa, females of aquatic bugs tend to be larger than the males (Truxal 1949). Some of the species descriptions provide the maximum and minimum length of the body for both females and males. The maximum values are often larger for females, but there is also a significant overlap between the minimum and maximum ranges of the sexes. Statistical comparisons, however, have been made on a few species only (Svensson et al. 2000; Nosil 2001; Bakonyi et al. 2016). Therefore, statistically proven differences between the body sizes of the two sexes are largely unknown.

SSD in Nepomorpha has not yet been systematically investigated except for the Belostomatidae (Ohba et al. 2019). Iglesias et al. (2010, 2012) investigated SSD in the family Belostomatidae. The body length of five *Belostoma* species showed no sexual difference, but male‐biased SSD was found in their middle and hind leg segments. In contrast, *Lethocerus annulipes* (of the same family) exhibits a female‐biased SSD but no difference in leg segments, which is likely related to differences in offspring care between the two genera (Iglesias et al. 2012; Ohba et al. 2019).

The species of the Belostomatidae possess unique mating systems. Females of Lethocerinae lay their eggs on shore plants, while females of Belostomatinae glue eggs on the backs of males. In both cases, the males take care of the eggs. Paternal care plays a role in maintaining oxygen and humidity in the environment of the eggs and thus in their survival and development (Ohba 2019). Therefore, females prefer and often compete for larger males who can provide better parental care and protection for the eggs (Tallamy 2001). The bigger, wider males of *Belostoma angustum* provide a larger surface area for oviposition, which may be a selection advantage (Iglesias et al. 2010; Lopes et al. 2020). According to Ohba et al. (2019), this phenomenon is common to the entire Belostomatinae. However, the extent to which male dorsum size increases male fitness is not yet fully understood (Lopes et al. 2020). A further selection criterion in *Lethocerus deyrollei* is that males mate several times with the same female to prevent sperm competition (Ichikawa 1989; Smith 1979). Despite this fragmentary information, we still do not fully understand the relationship between female preferences and male parental care in belostomatids (Ohba 2019).

Nepomorpha is a monophyletic taxon, a sister group to all other Heteroptera (Li et al. 2012; Weirauch et al. 2019). All Nepomorpha are aquatic, except for the Ochteroidea, which have adapted to a riparian mode of life (Wang et al. 2020). The transformation of the swimming legs into walking legs and the anal siphon in Nepidae enabled the development of a sit‐and‐wait predatory way of life, which allowed them to occupy new habitats and thus reduce competition (Wang et al. 2020). When a taxon changes its habitat on a macroevolutionary scale, it is often accompanied by a change in sexual dimorphism (Boisseau et al. 2020). Consequently, the colonisation of new habitats may also underlie the greater sexual differentiation of Ochteroidea and Nepidae.

Body size and SSD may be related. Rensch (1950) outlined a macroevolutionary trend in the relationship between body size and SSD in comparisons across closely related species. Rensch's rule (RR) states that larger species tend to have relatively larger males (relative to female size). In taxa where the male is the larger sex SSD increases with body size and, contrarily, in taxa where the female is the larger sex SSD tends to decrease with increasing body size (Meiri and Liang 2021). Some animal taxa exhibit an opposite trend called converse Rensch's rule (ConRR). Abouheif and Fairbairn (1997) found that the rule held broadly, especially in animal taxa where the male is the larger sex. Among insects, females are generally larger than males (female‐biased SSD), but the degree of size difference between the sexes varies across different taxa due to various evolutionary pressures (Blanckenhorn et al. 2007). Female‐biased SSD was observed in 72%–95% of species in seven orders of insects (Stillwell et al. 2010). Teder and Tammaru (2005) found that the SSD of 81.6% of 158 insect species investigated was female‐biased, and the SSD increased with the species' body size ConRR. The morphological plasticity of the larger sex, whether male or female, was greater than that of the other sex (Rohner et al. 2018).

The present study aims to answer three main questions. Firstly, we have examined the extent of SSD in 21 genera belonging to all five superfamilies of Nepomorpha (Corixoidea, Nepoidea, Ochteroidea, Notonectoidea and Naucoroidea; Wang et al. 2020). We assumed that, as in most insect groups, females represent the larger, longer‐bodied sex in all taxonomic groups. Secondly, we examine whether the SSDs of species inhabiting seven different habitat types differ from each other. The association of aquatic bug species to their habitat is well known (Hufnagel et al. 1999; Slimani et al. 2022). Their preferences depend on habitat characteristics such as biotope structure, predation pressure, availability of nutrient sources, population density and access to resources, among others. These factors may also impact the extent of species SSD. Hence, we hypothesised that the SSD of species in different habitats should differ. Finally, we examine the pattern of RR or ConRR at different taxonomical levels.

## 2 Methods

### 2.1 Data Collection and Processing

Female and male body length data were collected for 1195 species, subspecies and wing‐morphs belonging to five superfamilies, 11 families and 68 genera (for the complete dataset see Table S1). The total number of species (including subspecies and wing‐morphs) is 1097, representing about 45% of all known Nepomorpha. Body length was considered a proxy for body size. There is a close relationship between wing polymorphism and body size, as Young (2010) has shown in Notonectidae. Therefore, macropterous and micropterous morphs of the same species were considered separately. The database was supplemented with unpublished data obtained by G. Bakonyi in the Hemiptera Collection of the Hungarian Natural History Museum with an ocular micrometre using an Opton 475,052–9901 microscope and digital calliper ExtolPremium. The data and species names in Table S1 are presented as in the original articles. We retrieved the phylogenetic relationships from Wang et al. (2020) to classify our taxa. If there was any doubt about which genus a species belonged to, we followed the Integrated Taxonomic Information System (ITIS). Where different authors have reported body sizes for the same species, the data have been averaged. Calculations were performed using the averages. If only one data point was provided, it was used as a proxy for the average. Often only the minimum and maximum data of the species' body length was available, in which case the arithmetic mean of these was calculated and used further.

Some species descriptions provide sample size, mean and minimum and maximum data of body length. We compared how sample means and the averages calculated from the minimum and maximum data predict each other. A total of 116 and 121 species were involved for females and males, respectively (Table S2). These data were supplemented with our unpublished data, and thus, the comparison was based on 142 species. The differences between the two indices were only a few % in most cases and never exceeded 10%. Therefore, averages based on the minimum and maximum data were considered a reliable proxy for the sample mean.

There are several methods to measure the SSD of species (Meiri and Liang 2021). In this study, the sexual dimorphism index (SDI) most commonly used in ecological studies was used; SDI = (length of female/length of male)–1, negative when males are larger than females and positive when females are the larger sex. This is a slightly modified version of the index described by Lovich and Gibbons (1992).

### 2.2 Data Grouping by Habitat

Aquatic bugs inhabit a wide range of habitats and can be grouped according to their life history (mainly swimming, feeding and breathing) and behaviour. For example, Menke (1979) has compiled a key for aquatic and semiaquatic Hemiptera in California based on their habitat and species habits (way of life). Following this line of thinking, we apply seven categories of freshwater habitats (Table S3). (1) Riparian habitat is the transitional area between aquatic and terrestrial ones, found along the edges of lakes, ponds, rivers, streams and other water bodies. The soil moisture content is high. The diversity of plants and animals is also high, as is usually the case in ecotones. It is home to secondary terrestrial Nepomorpha species (e.g., *Ochterus*, *Nerthra*). (2, 3) The littoral habitat is the interface between the water body and the shore of both standing and running waters. This area is submerged in water where sunlight penetrates to the bottom, allowing diverse aquatic plants to grow. It is a well‐structured habitat for insects, offering abundant resources like food, shelter and breeding grounds. This habitat has been divided into two parts. (2) The area close to the shore with floating plants is home to species that are elongated, slender and poor swimmers, foraging as sit‐and‐wait predators (e.g., *Nepa*, *Ranatra*). This habitat is called littoral(c) (c: close to the shore). (3) The area of the littoral habitat towards open water with submersed plants is home to species with streamlined bodies, which are good swimming predators, temporarily moving away from the shore (e.g., *Belostoma*, *Lethocerus*). This habitat is called littoral(o) (o: towards open water). The parts of standing waters outside the littoral habitat towards the interior of the water body where the animals can move from the surface to the bottom are called open‐water or (4) pelagic habitats. Many Corixidae species live in pelagic habitats, although some are also found in other habitats. (5) Benthic habitat refers to bottom sediment, which serves as a nursery, refuge and foraging area for aquatic bugs. Small‐bodied species are living here (e.g., Micronecta, Tenagobia). (6) Nektonic habitats constitute the surface and the uppermost zone of water bodies, where *e.g., Notonecta* and *Buenoa* species live. Finally, riverine, rocky habitats with deeper areas as pools and shallower areas with swiftly flowing, turbulent water are called (7) riffles. The substrate is usually dominated by cobble or gravel. This is the home of the creeping water bugs (e.g., *Ambrysus*, *Aphelocheirus*, *Limnocoris*, *Temnocoris*). We are aware that, like all habitat classifications, ours is not perfect. Moreover, there can be differences in habitat preferences between congeneric species. However, we assume that the number of misclassified genera is small given that the life histories of related species tend to be similar.

### 2.3 Phylogenetic Tree Construction

**FIGURE 1:**
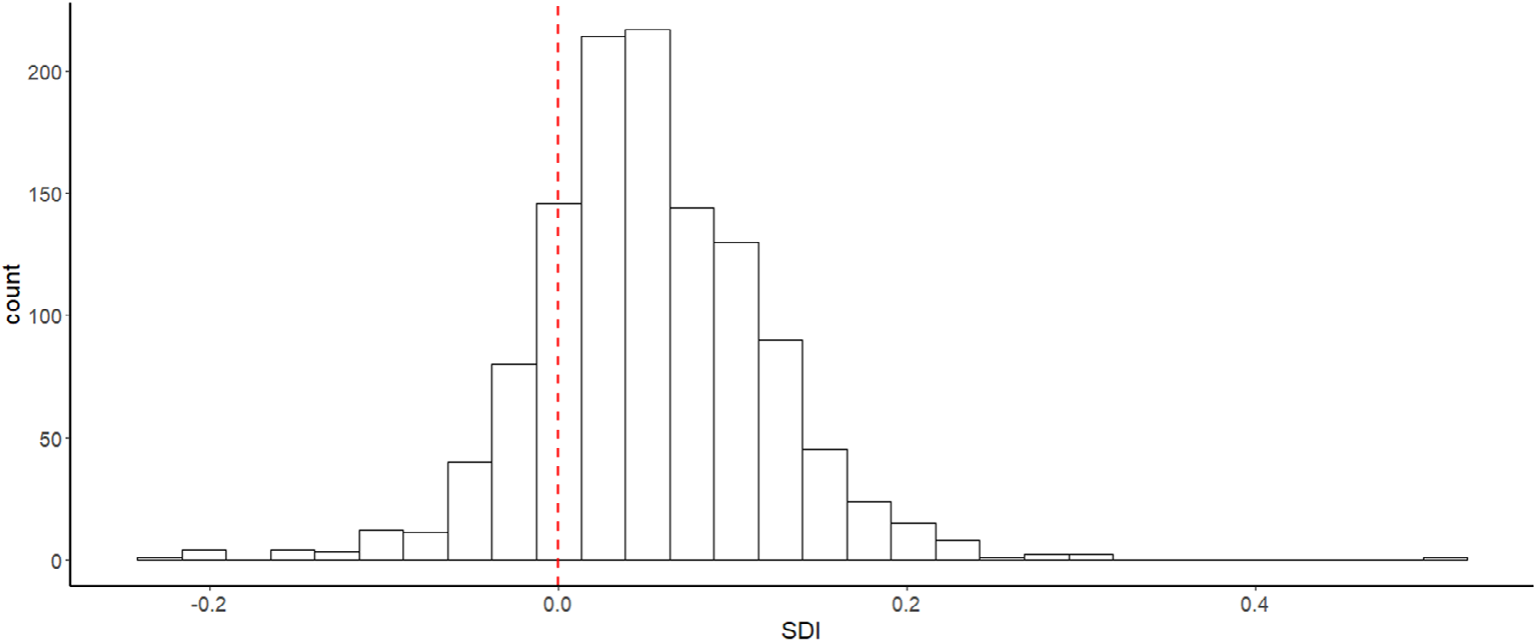
Sexual dimorphism index (SDI) frequency distribution of 1195 Nepomorpha aquatic bug species. Sexual size dimorphism was calculated based on body length. Positive values indicate female, negative values indicate male biased dimorphism. Red dashed line: SDI = 0.

A phylogenetic tree was constructed for the 21 genera with the iTOL v6 software (Letunic and Bork 2024). Node positions and branch lengths were based on figure 1 by Wang et al. (2020). The location of the genus on our tree is taken to be the same as the location of the species in that genus by Wang et al. (2020). If more than one species of a genus was present in the figure, the average of branches was taken. If there were no species of a given genus, its name was placed at the nodus of the closest genus or genera. The position of the genus *Sigara* was determined according to Ye et al. (2023), *Curicta* to Ye et al. (2020) and *Asthenocoris* and *Ambrysus* to Schuh and Slater (1995).

### 2.4 Statistical Analysis

To determine whether the difference between the body length and SDI values of the sexes is significant or not, we used a two‐sample t‐test. We examined whether habitat has an effect on SDI and RMA slope values. For this aim, we performed a one‐way ANOVA analysis. The assumptions for the tests we checked with a Q‐Q plot (normality of the residuals), F‐test (homoscedasticity of the residuals), Grubbs test and Dixon's test (outliers). Omega squared (ω^2^) was used as a measure of effect size for the ANOVA main effect. If the ANOVA main effect was significant, a Tukey post hoc test was performed. In this case, the 95% confidence intervals for the differences in group means were regarded as effect size. There are large differences in body length between Nepomorpha species. In order to compare the variability of body length datasets with considerably different means, we calculated coefficients of variation (cv%). Evaluation for the equality of cv% was considered using the cvequality test (Marwick and Krishnamoorthy 2019).

In small datasets, regression models have little or no statistical power. That is why we performed sample size estimation for regression analysis using GPower version 3.1.9.7 (Faul et al. 2007). The required sample size was determined with a significance criterion of *α* = 0.05 and power = 0.80 for each taxon. We excluded from regression analyses genera for which the number of available data did not meet the significance criteria. We performed OLS regression in two cases. Firstly, we were interested in whether there is a relationship between the RMA slope values of the genera and the SDI. Secondly, we examined the regression between the proportion of female species in a genus and the corresponding mean SDI values.

To test RR, we performed a reduced major axis (RMA) regression. The body length of females was plotted on the *x*‐axis and that of males on the y‐axis. The null hypothesis was that the slope of the regression line was 1. The body length data were log_10_ transformed to improve the normality of the data distribution. The correlation coefficient was regarded as effect size. A slope greater than 1 indicates a pattern consistent with RR, while a slope less than 1 indicates ConRR. A difference from slope 1 was considered significant if both 95% confidence limits were either below or above 1. Analyses were made on the infraorder, superfamily, family and genus level. We performed all analyses with the PAST4 statistical software (Hammer et al. 2001) except for the evaluation of the equality of coefficients of variation and sample size estimation for regression analysis.

## 3 Results

### 3.1 Sexual Size Dimorphism

The body size of Nepomorpha species is quite variable. In our database, the largest species *Lethocerus maximus* (100.35 mm) was two orders of magnitude larger than the smallest *Micronecta clavata* (1.24 mm). The average size of the genera also often differed markedly (Table 1). However, the body size of congeneric species was relatively uniform. Only the species in the Belostoma genus had little above 40 cv%. In most other cases, a moderate variability, between 10 and 30 cv%, was found. Remarkably similar cv% values were obtained for females and males of the same species (Table 1). The difference in the cv% between the sexes was, on average, 0.4%, and the highest value was only 3.0% (*Nerthra*).

**TABLE 1:** Body length data for species in different genera.

	*N*	Species average (mm ± SD)	cv%	Female (mm ± SD)	cv%	Male (mm ± SD)	cv%	*p*
*Ambrysus*	30	9.88 ± 2.1	21.6	10.18 ± 2.2	21.2	9.57 ± 2.1	22.3	0.981
*Anisops*	100	6.44 ± 1.4	21.6	6.52 ± 1.4	21.1	6.36 ± 1.4	22.6	0.824
*Aphelocheirus*	82	7.99 ± 1.75	20.6	8.08 ± 1.6	20.2	7.89 ± 1.7	21.3	0.882
*Asthenocoris*	12	7.47 ± 0.5	7.2	7.57 ± 0.6	7.3	7.37 ± 0.5	7.2	0.922
*Belostoma*	76	24.67 ± 10.2	41.3	24.85 ± 10.3	41.4	24.49 ± 10.1	41.2	0.991
*Buenoa*	81	6.63 ± 1.5	22.7	6.76 ± 1.5	22.1	6.50 ± 1.5	23.6	0.920
*Curicta*	18	22.19 ± 4.3	19.6	23.4 ± 4.4	18.9	20.99 ± 4.3	20.4	0.982
*Enithares*	65	9.54 ± 1.9	20.0	9.48 ± 1.8	19.0	9.59 ± 2.1	21.4	0.720
*Gelastocoris*	18	7.53 ± 0.7	9.1	7.95 ± 0.7	9.3	7.11 ± 0.7	9.3	0.740
*Hesperocorixa*	12	7.92 ± 1.9	24.1	8.21 ± 2.0	24.0	7.64 ± 1.9	24.2	0.942
*Laccotrephes*	17	30.04 ± 8.7	28.9	30.85 ± 8.7	28.1	29.23 ± 8.8	30.0	0.997
*Lethocerus*	23	67.50 ± 12.9	19.1	70.35 ± 13.7	19.5	64.65 ± 12.5	19.3	0.982
*Limnocoris*	72	7.89 ± 1.5	19.2	8.03 ± 1.6	19.6	7.75 ± 1.5	18.9	0.796
*Micronecta*	79	2.29 ± 0.7	28.7	2.32 ± 0.7	29.7	2.27 ± 0.6	27.9	0.557
*Nerthra*	61	8.33 ± 1.5	18.4	8.66 ± 1.5	17.2	7.99 ± 1.6	20.2	0.803
*Notonecta*	26	13.02 ± 2.4	18.2	13.38 ± 2.7	19.9	12.66 ± 2.2	17.0	0.767
*Ochterus*	57	4.51 ± 1.0	12.8	4.72 ± 0.6	13.5	4.30 ± 0.6	12.9	0.386
*Ranatra*	87	32.30 ± 6.9	21.4	33.81 ± 7.5	22.0	30.79 ± 6.5	21.1	0.897
*Sigara*	51	6.15 ± 0.9	15.2	6.33 ± 0.9	14.9	5.98 ± 0.9	15.7	0.996
*Tenagobia*	34	2.70 ± 0.6	21.9	2.78 ± 0.6	21.6	2.62 ± 0.6	22.4	0.941
*Trichocorixa*	22	4.04 ± 0.5	11.4	4.20 ± 0.5	11.5	3.88 ± 0.5	11.8	0.805

*Note:* Mean body sizes (±SD) and coefficients of variation (cv%). *p* = significance between female and male cv% (cvequality test).

Abbreviation: *N*, Number of species.

Across all species, females were larger in 942 cases (78.8%), while males were larger in 196 cases (16.4%) and no differences were found in 57 cases (4.8%), disregarding statistical significance. For 142 species of 12 genera, however, data allowed us to analyse the differences between body lengths statistically (Table S4). In 44.4% of these cases, there was no significant difference between the two sexes, while in 55.6% females were significantly larger. No species had significantly larger males than females (Table 2). There were substantial differences among the genera in this respect. In the genus *Martarega*, *Nerthra* and *Ranatra*, females were significantly larger than males in all species studied. In contrast, no significant difference was found between the sexes in any *Diplonychus* species (Table S4). For the other eight genera, there were varying proportions of species with significant differences between the sexes and those without. Moreover, in some species, SSD differed between different populations, as exemplified by *Buenoa albida* , *B. antigone antigone*, *B. gracilis* and *B. scimitra* (Table S4).

**TABLE 2:** Number (frequency) of female‐, equal and male‐biased species in those genera where appropriate data for *t*‐test calculation were available for at least 5 species.

Genus	*N*	*F* > *M*	*F* = *M*	*F* < *M*
*Belostoma*	5	2 (40.0)	3 (60.0)	0 (0)
*Buenoa*	33	25 (75.8)	8 (24.2)	0 (0)
*Corixa*	5	1 (20.0)	4 (80.0)	0 (0)
*Diplonychus*	6	0 (0.0)	6 (100)	0 (0)
*Laccotrephes*	5	3 (60.0)	2 (40.0)	0 (0)
*Martarega*	8	8 (100)	0 (0.0)	0 (0)
*Micronecta*	30	7 (23.3)	23 (76.7)	0 (0)
*Nerthra*	9	9 (100)	0 (0.0)	0 (0)
*Notonecta*	18	6 (33.3)	12 (66.7)	0 (0)
*Ranatra*	5	5 (100)	0 (0.0)	0 (0)
*Sigara*	13	9 (69.2)	4 (30.8)	0 (0)
*Tenagobia*	5	4 (80.0)	1 (20.0)	0 (0)
Total	142	79 (55.6)	63 (44.4)	0 (0)

Abbreviation: *N*, Number of species.

**FIGURE 2:**
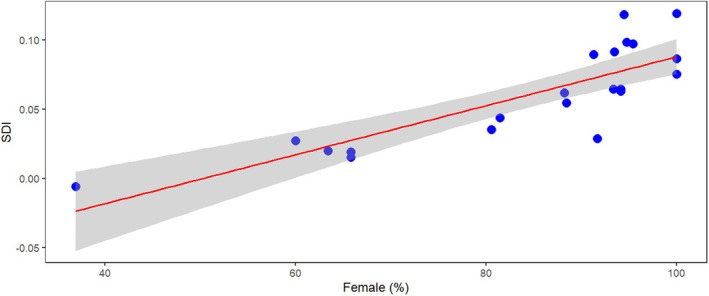
Covariation of the proportion of species where females are larger than males and mean SDI of each genus (female % of Nepomorpha). There is a significant positive relationship between the two factors. The grey area depicts the 95% confidence interval of the regression.

Overall, Nepomorpha species exhibit a narrow range of SDI. The species‐specific body length SDI ranged from −0.24 to 0.5. Most of the data fall in the positive range, indicating that body length in most species is female‐biased (Figure 1). Except for *Enithares*, the average values are always positive at the genus level, showing that body size in these genera is on average female‐biased (Table S5). All *Curicta*, *Hesperocorixa* and *Trichocorixa* species were female‐biased. As the proportion of female‐biased species in a genus increased, so did the average SDI value of the genus (regression: *t* = 6.57, *r* = 0.833, *p* < 0.001; Figure 2).

### 3.2 SDI in Various Habitats

**FIGURE 3:**
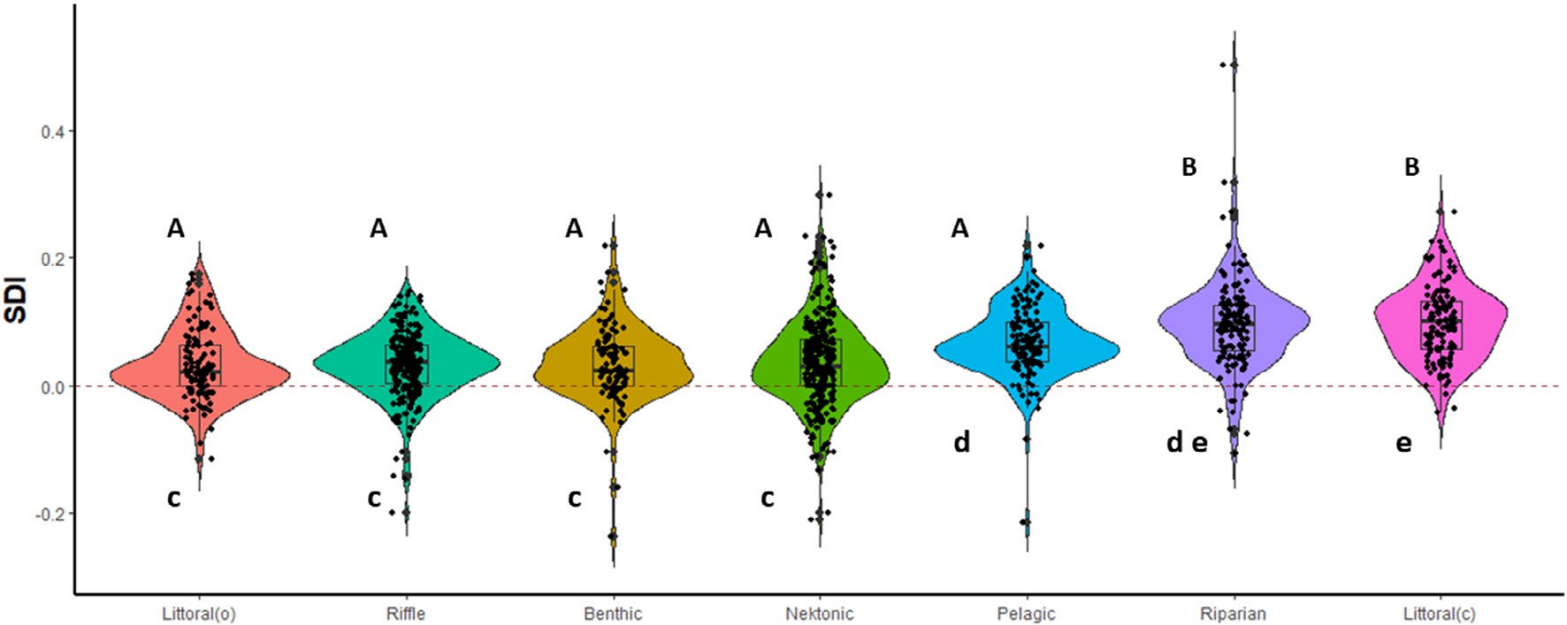
Distribution of SDI values in the seven habitats on violin plots with the addition of a boxplot and jitter (raw data). Capital letters refer to SDI values, small letters to coefficients of variation. Different letters indicate statistically significant differences at the minimum *p* < 0.05 level. Red dashed line: SDI = 0.

There was a significant effect of habitat on SDI values (ANOVA: *F*_6,1188_ = 37.1, *p* < 0.001, ω^2^ = 0.154). Based on the post hoc analysis (Table S6), habitats could be classified into two groups. Species found in the riparian habitat (Ochteridae, Gelastocoridae) and poorly swimming species regularly present in the littoral habitat close to the riverbanks or lakeshores (Nepidae) were grouped together (Group1: riparian + littoral(c)). These species exhibit the highest SDI values (Figure 3). The SDI values of the species in all other habitats are similar (Group2: pelagic + benthic + littoral(o) + nektonic + riffle). The SDI values of the two groups differed at the *p* < 0.001 level except for the pelagic group, whose SDI values differed at the *p* < 0.002 level from Group1. The effect sizes were significant in all groups because the lower bounds of the confidence intervals of the medians were greater than zero (Figure 3). Coefficients of variation of genera SDIs in various habitats were also different (D'AD = 65.98, *p* < 0.001). Moreover, coefficients of variation of SDIs for riparian, littoral(c) and pelagic habitats were significantly lower than those for other habitats (Table S6).

### 3.3 Rensch's Rule

**FIGURE 4:**
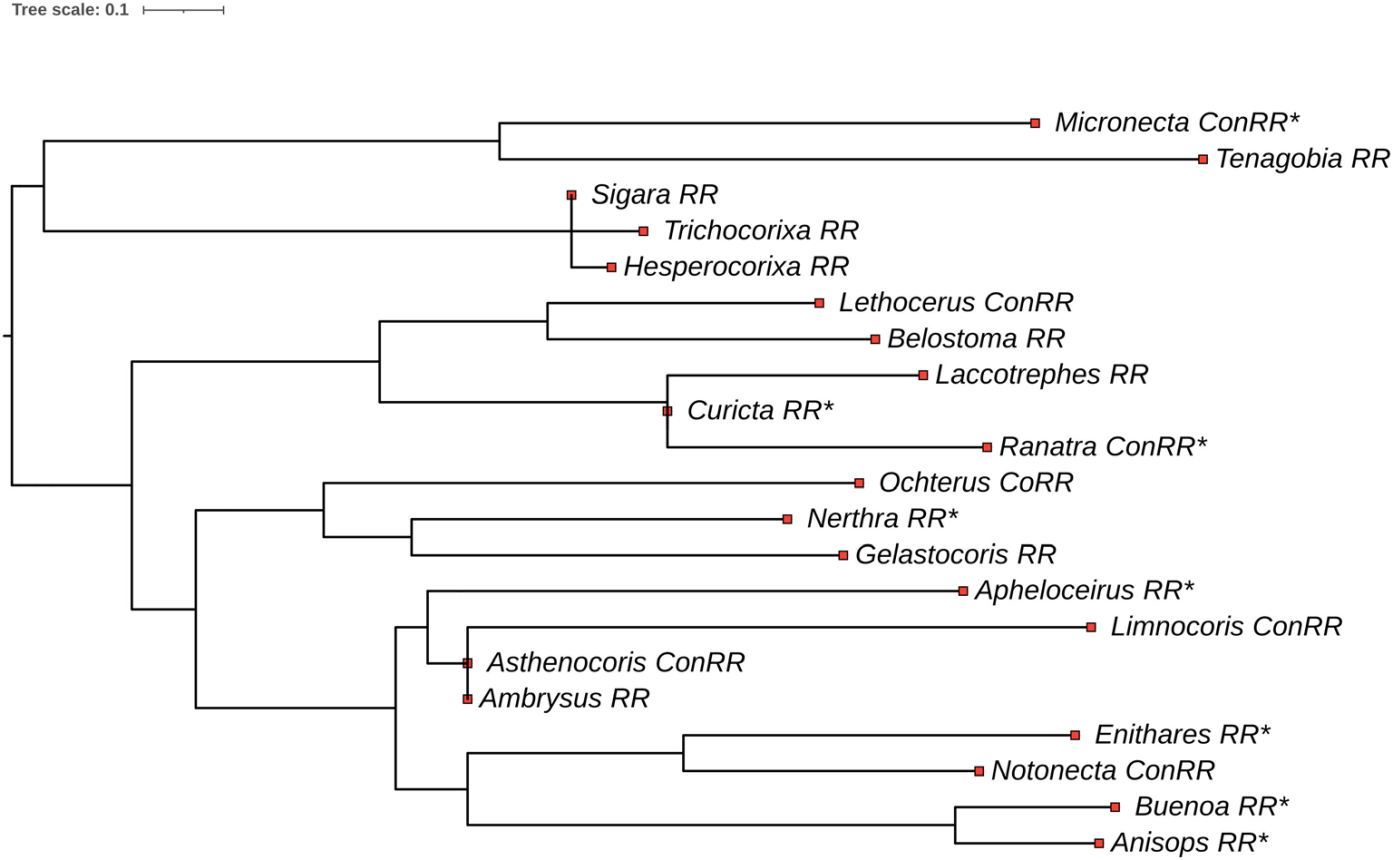
Phylogenetic tree for the 21 Nepomorpha genera. Node positions and branch lengths were based on Figure 1 in Wang et al. (2020). The red points show the position of the genus after the point on the phylogenetic tree. An asterisk indicates that the RR or ConRR is statistically significant for the genus.

Our RMA regressions performed for four taxonomic levels (infraorder, superfamily, family, genus) resulted in different slopes (Table 3). SSD of the Nepomorpha infraorder followed ConRR. This means that the difference in size between males and females becomes more pronounced as the species' size increases. As we look at lower taxonomic levels, we see differences between taxa. In the case of superfamilies, RR is verified for Nepoidea and Corixoidea. We found diverse patterns at the family level. Isometry was found for the families Aphelocheiroidea, Naucoridea, Nepidea and Ochteridea. The families Aphelocheiridae, Gelastocoridae and Notonectidae conformed to the RR, while the families Belostomatidae and Micronectidae follow the ConRR. We had enough data for 21 genera to perform a reliable regression analysis. For 14 out of the 21 genera, the MRA slopes did not differ significantly from one. We found significant RR in six genera (*Anisops*, *Aphelocheirus*, *Buenoa*, *Curicta*, *Enithares*, *Nerthra*) and ConRR patterns in two (*Micronecta*, *Ranatra*). It is apparent that in the Nepomorpha infraorder, the scaling relationships at multiple taxonomic levels show different figures for the validity of RR (Table 3). Further, we obtain a diverse pattern in terms of RR when the genera are plotted on a phylogenetic tree (Figure 4). In most cases, we found that closely related genera show different trends, except for *Enithares*, *Buenoa* and *Anisops*. These genera are obeying RR and belong to the same family. So, we can assume similar evolutionary causes underlying their SSD.

**TABLE 3:** Results of the RMA regressions performed for infraorder, superfamily, family, and genus.

Infraorder	*N*	*r* ^2^	Slope	Intercept	95% CI lower–upper	Significance	Trend
Nepomorpha	1195	0.994	0.994	−0.016	0.990–0.999	< 0.001	**ConRR**
**Superfamily**							
Aphelocheiroidea	82	0.905	1.073	−0.077	0.996–1.123	< 0.001	RR
Corixoidea	249	0.991	0.972	−0.004	0.962–0.982	< 0.001	**ConRR**
Naucoroidea	167	0.973	1.009	−0.024	0.984–1.038	< 0.001	RR
Nepoidea	246	0.983	0.980	0.003	0.962–0.995	< 0.001	**ConRR**
Notonectoidea	312	0.962	1.024	−0.034	0.983–1.074	< 0.001	RR
Ochteroidea	137	0.965	1.015	−0.052	0.982–1.048	< 0.001	RR
**Family**							
Aphelocheiridae	82	0.968	1.057	−0.059	1.012–1.097	< 0.001	**RR**
Belostomatidae	112	0.994	0.969	0.969	0.952–0.985	< 0.001	**ConRR**
Corixidae	136	0.971	1.009	−0.034	0.979–1.039	< 0.001	RR
Gelastocoridae	80	0.901	1.130	−0.160	1.037–1.220	< 0.001	**RR**
Micronectidae	113	0.960	0.953	0.004	0.925–0.983	< 0.001	**ConRR**
Naucoridae	167	0.973	1.009	−0.024	0.984–1.0379	< 0.001	RR
Nepidae	134	0.969	1.017	−0.065	0.987–1.049	< 0.001	RR
Notonectidae	301	0.963	1.065	−0.071	1.036–1.095	< 0.001	**RR**
Ochteridae	57	0.759	0.960	−0.013	0.844–1.074	< 0.001	ConRR
**Genus**							
Ambrysus	30	0.972	1.017	−0.043	0.918–1.089	< 0.001	RR
Anisops	100	0.920	1.070	−0.068	1.008–1.121	< 0.001	**RR**
Aphelocheirus	82	0.968	1.057	−0.059	1.012–1.097	< 0.001	**RR**
Asthenocoris	12	0.903	0.993	−0.006	0.758–1.153	< 0.001	ConRR
Belostoma	76	0.994	1.004	−0.012	0.984–1.022	< 0.001	RR
Buenoa	81	0.954	1.072	−0.077	1.024–1.128	< 0.001	**RR**
Curicta	18	0.973	1.085	−0.164	0.964–1.174	< 0.001	RR
Enithares	65	0.941	1.148	−0.141	1.071–1.238	< 0.001	**RR**
Gelastocoris	18	0.878	1.003	−0.051	0.738–1.124	< 0.001	RR
Hesperocorixa	12	0.990	1.007	−0.038	0.957–1.076	< 0.001	RR
Laccotrephes	17	0.982	1.056	−0.108	0.964–1.120	< 0.001	RR
Lethocerus	23	0.890	0.992	−0.023	0.850–1.092	< 0.001	ConRR
Limnocoris	72	0.970	0.974	0.009	0.930–1.017	< 0.001	ConRR
Micronecta	79	0.963	0.955	0.007	0.919–0.990	< 0.001	**ConRR**
Nerthra	61	0.901	1.132	−0.160	1.026–1.226	< 0.001	**RR**
Notonecta	26	0.921	0.915	0.072	0.756–1.060	< 0.001	ConRR
Ochterus	57	0.759	0.960	−0.013	0.838–1.064	< 0.001	ConRR
Ranatra	87	0.949	0.946	0.042	0.909–0.985	< 0.001	**ConRR**
Sigara	51	0.929	1.068	−0.080	0.989–1.149	< 0.001	RR
Tenagobia	34	0.956	1.038	−0.043	0.976–1.103	< 0.001	RR
Trichocorixa	22	0.862	1.027	−0.052	0.717–1.163	< 0.001	RR

*Note:* The *p* values for regression coefficients were < 0.001 in all cases. *N*, Number of species, trend: The validity of Rensch's rule, RR, Rensch's rule is valid; ConRR, Converse Rensch's rule is valid. Bold = both 95% confidence limits are below or above 1 for the full range (significant difference from 1).

### 3.4 Slope Values by Habitat and SDI

**FIGURE 5:**
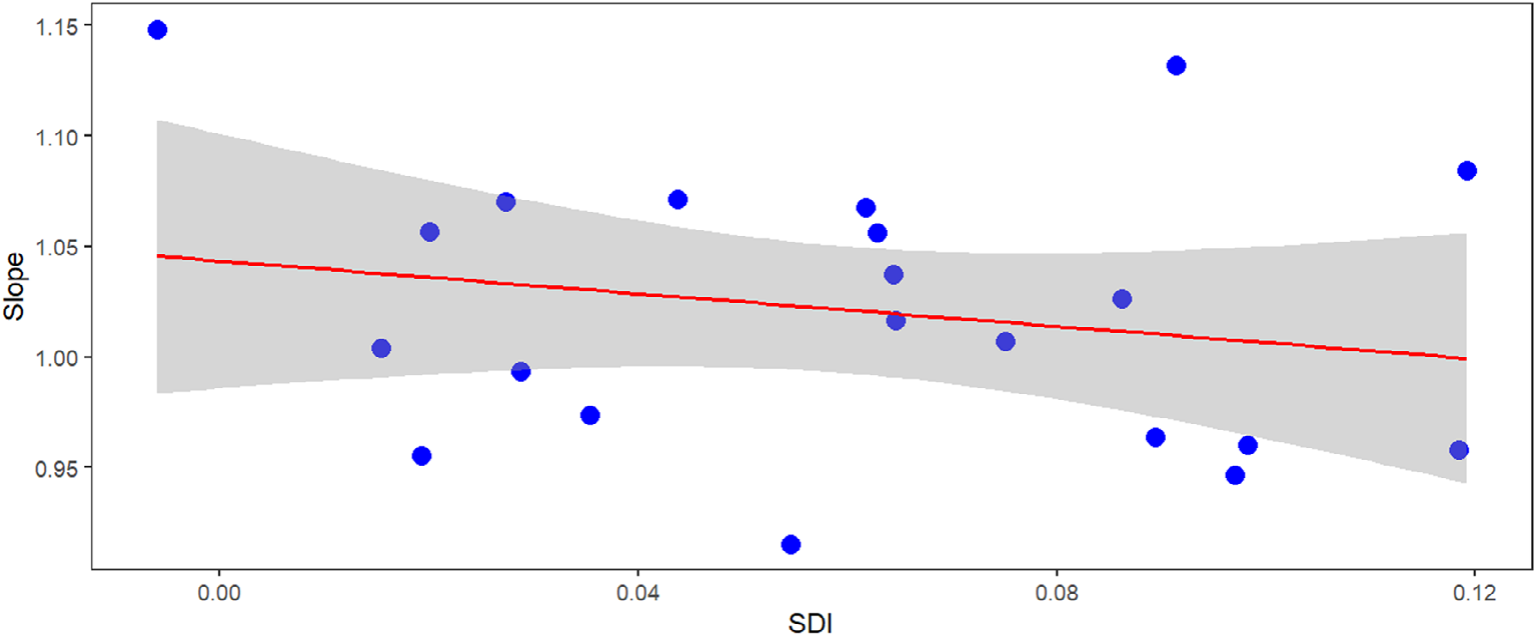
The relationship between the allometric slopes (of major axis regression lines between male and female body length) and the mean SDIs of the 21 genera. The grey area depicts the 95% confidence interval of the regression.

We compared the slopes of the RMA of species belonging to different habitats. No differences were found by habitat (ANOVA: *F*_6,14_ = 0.24, *p* = 0.957, *ω* ^2^ = 0.0). We also examined whether there was a relationship between the RMA slope values of the genera and the SDI. However, the regression was not significant (*t* = 0.934, *p*_(slope)_ = 0.362, *r* ^2^ = 0.044); (Figure 5).

## 4 Discussion

### 4.1 Monomorphic Nepomorpha Species Are Quite Common

Like other insects (Blanckenhorn 2000; Stillwell et al. 2010), most species (in about 80% of our sample) of aquatic bugs have larger females than males. There are still noteworthy deviations from this main trend, however. Just over half of the species showed statistically significant sexual differences in body length of the sexes in our study. Since the sexual difference in body size between females and males can vary significantly between populations (Bakonyi et al. 2016; Nieser 1967, 1975; but see Sites and Willig 2000), differences at the species level can only be detected using large samples. In most cases, unfortunately, sample sizes are limited, and often only minimum/maximum data are available, thus, significant differences between the sexes cannot be calculated. Our statistical evaluation shows that there are actually fewer female‐biased species and more monomorphic species than the minimum/maximum data would suggest. This is supported by the fact that the species‐specific SDI falls within a narrower range than similar values, for example, copepods (Hirst and Kiørboe 2014), orthopteroid insects (Bidau et al. 2016), fishes (Horne et al. 2020) and lizards (Liang et al. 2022). At the genus level, the coefficient of variation in body lengths of females and males was not significantly different and the absolute values differed by only a few percent. By contrast, Kojima (2019) for beetles and Bidau et al. (2016) for orthopteroid orders found large differences in the coefficient of variation between the sexes. Our assumption that females are longer‐bodied in most aquatic bug species is falsified.

### 4.2 Habitat Affects Sexual Dimorphism

We found that habitat type is associated with the degree of sexual dimorphism in aquatic bugs. The SDI of species living on the riparian and in still or slow‐moving waters adjacent to the shore (Group1) is higher than that of the other five habitats (Group2). Furthermore, the coefficient of variation for the species in Group2 varies more than for Group 1. This pattern suggests that environmental and ecological factors act in a sex‐specific way in some habitats and less or not in others. Species in different habitat types are subject to distinct physical structures, resource availability, predation pressure and competition. All these factors can affect the SSD to varying degrees (Butler et al. 2000; Teder and Tammaru 2005) and are consistent with the view that, in addition to sex‐specific sexual selection, ecological processes can have a significant impact on sexual dimorphism in body size (Shine 1989; Chown and Gaston 2010; Winkler et al. 2024). We assume that this pattern is primarily a consequence of the high structural diversity of riverbank and lakeshore habitats, where animals can easily find hiding places to wait for prey, and also the high diversity and abundance of food species in the riparian (Paetzold and Tockner 2005; Ramey and Richardson 2017) and littoral habitat (Walseng et al. 2006; Heino 2008; Maia‐Barbosa et al. 2008). Moreover, it cannot be ruled out that natural selection for mobility also played a role in the emergence of the two groups (Byers et al. 2010; Barske et al. 2011), although, to our knowledge, there are no data to support this hypothesis for Nepomorpha.

Habitat‐related SSD was repeatedly detected in vertebrates, e.g., in fishes (Horne et al. 2020), *Anolis* lizards (Butler et al. 2000), voles (García‐Navas et al. 2016), ungulates (Pérez‐Barbería et al. 2002) and horseshoe bats (Wu et al. 2018), but not in *Eleutherodactylus* frogs (Dugo‐Cota et al. 2022). We have much less information about insects. Contrary to our results, habitat properties did not affect sexual dimorphism in either ambush bug *Phymata americana* (Punzalan et al. 2008) or Iberian grasshoppers (Acrididae) (García‐Navas et al. 2017). However, the sexual dimorphism of butterflies in the understorey of Amazonian rainforests was greater than that of butterfly species in the canopy due to the more complex physical structure of this stratum and greater predation pressure (Graça et al. 2017). Biró et al. (2024) found that the degree of sexual dimorphism in eight *Niphargus* species differs between habitats in traits related to sensing, feeding, oxygenation and crawling. They found that the magnitude of natural (environmental) selection was comparable to the magnitude of sexual and fecundity selection.

Group1 species are all sit‐and‐wait predators, with the exception of Ochteridae. In contrast, Group2 species are active foragers. Therefore, it is reasonable to assume that feeding behaviour also plays a role in the development of SSD differences between the two groups. According to Schoener's (1969) optimal feeding model, a larger difference in SSD is expected for sit‐and‐wait predators than for active foraging predators that hunt in other ways. Schoener (1969) found the model to be valid for birds, Butler et al. (2000) for Antillean *Anolis* lizards and Wu et al. (2018) for horseshoe bats (Rhinolophidae). However, no such association was found for crab spiders (Thomisidae) (Rocha and Gawryszewski 2024) and British and Irish spiders (Prenter et al. 1997). Our results are consistent with the prediction of Schoener's model. As regards the potential impacts of habitat and food sources on Nepomorpha species, we found the highest SSD along the riparian habitat and in waters close to the shore (Group1). The Nepomorpha species present here are sit‐and‐wait predators (except Ochteridae). These habitats are structurally diverse (Zohary and Gasith 2014) and biodiverse (Strayer and Findlay 2010; Ramey and Richardson 2017). Both features make them optimal for sit‐and‐wait predators because they can easily hide from their own predators, find a suitable place for hunting and are more likely to find optimal‐size prey within the range of their predatory legs.

Information on mate choice is not available for species in Group1 habitats (the species referred to in this paragraph all belong to this group). Specific courtship and before‐mating behaviours were not observed in *Gelastocoris oculatus oculatus* (Brown and McPherson, 1994), *Nerthra nepaeformis* (Keith and McKevan, 1942), *Curicta howardi* (Keffer and McPherson, 1993), *Ranatra vicina* (Tawfik and Awadallah, 1975) and *Laccotrephes maculatus* (Khandelwal and Gupta, 2017). The lifting of the female's abdomen, the vibration of the male's hind leg and the male's front leg touching the female during the precopulatory phase have been observed in a few cases in *Nepa hoffmani* (Hyun et al. 2024). During mating, males hold females firmly with their legs, especially with their front legs. The second and third pairs of legs may also play a role in fixing the female, but they are more responsible for holding the mating animals in surrounding solid objects (Tawfik and Awadallah 1975; Keffer and McPherson 1993). Hyun et al. (2024) found that *N. hoffmani* males with longer legs guarded females for longer periods.

When mating, males of *Ranatra vicina* slowly slide backwards on the female until the copulatory organs can join (Tawfik and Awadallah 1975). With this behaviour, longer first pairs of legs are obviously advantageous because they can hold even the larger female firmly. No differences in other traits were found between the sexes of *Ranatra* species. Sexual differences in the metabolic rate and physical activity in *R. linearis* do not appear to be significant for the development of SSD. Oxygen consumption per unit of body weight (Waitzbauer 1976) and breathing frequency (intervals between two successive visits on the water surface) (Cloarec 1988, 1996) were not different between the sexes. Furthermore, the attack rate of females and males was not different in *R. dispar* (Bailey 1986). Based on the mentioned findings about the mating behaviour, morphology and physiology of the species in the habitat Group1, we hypothesise that mating behaviour does not play an important role in the development of SSD in these species, but selection for male foreleg length does. Low SDI coefficient of variation values indicate that the environmental factors relevant for SSD are fairly balanced in habitats on both sides of the shore, in the riparian and close‐to‐shore habitats.

The pelagic habitat is home to most species of the family Corixidae. It is a monophyletic group (Ye et al. 2023), characterised by a triangular unsegmented rostrum (Hungerford 1948; Nieser 1970). The foreleg tarsus (pala) is commonly widened (Stonedahl and Lattin 1986) and males have characteristic, species‐specific pegs (Bakonyi 1984). Both morphological features serve to enable males to hold females tightly during mating. Meanwhile, a ridged, flat plate (strigil) on the sixth abdominal tergite of the male helps the exchange of air between the members of the pair (Popham et al. 1984a). Acoustic communication (stridulation) plays a role in mating in corixids, as in many other Nepomorpha taxa (Polhemus 1994). Although this issue has been studied relatively extensively in corixids, the results are variable and frequently contradictory. Their importance and specific role in reproductive behaviour are not clearly understood (Aiken 1985).

The extent of SDI in corixids, compared to other taxa, depends on the concerted action of several factors and causes, as illustrated by the example of *Sigara falleni* (Candolin 2004, 2005). Females prefer males with a larger body mass and a larger pala. These traits depend on the level of juvenile conditions. In the male–male competition, however, the individuals with smaller pala are favoured, because the cost of a large pala is high and the success of male–male competition depends on the body mass of the adults and good nutritional conditions in adulthood. The difference in body size between the sexes is therefore the result of opposite selection forces (Candolin 2004, 2005). Since pala has an opposite role in female mate choice and the male–male competition, we hypothesise that environmental factors, primarily food availability, influence the outcome of selection and the relatively high degree of SDI. This claim is supported by the finding that in *Callicorixa vulnerata* , where males do not have pala, fecundity selection is responsible for the increase in female body size (Nosil 2001). Unlike the other Nepomorpha taxa, corixids are generally omnivorous (except for the predatory subfamily Cymatiainae); (Hädicke et al. 2017) and their diet composition can vary seasonally (Bakonyi 1978). It is also remarkable that closely related species and even the sexes can differ in the composition of their diet (Popham et al. 1984b). Therefore, the development of SDI in corixids should be studied in the context of food quality and quantity. Most species are omnivorous, perhaps to provide optimal energy to reproduce under varying nutritional conditions.

There are some explanations why the SDI values of Group2 species are lower than those of Group1 species. Firstly, one reason for sexual dimorphism is the different ways in which the sexes interact with the environment (Slatkin 1984). This consequence may be different habitat choices of the sexes. However, in a mesocosm experiment, females and males of *Notonecta glauca* and *N. obliqua* were equally selective for habitats (Šigutová et al. 2022). Secondly, Svensson et al. (2000) hypothesise intersexual competition in food and habitat choice between the sexes of *N. lutea* , but not in the case of four other *Notonecta* species. Thirdly, Belostomatidae has specific courtship, mating and offspring care systems. The most important manifestations of these systems are the back‐brooding and emergent‐brooding behaviour (Ohba 2019). Polyandry is common, which reduces the degree of the SSD (Lopes et al. 2020). Iglesias et al. (2012) found selective forces shifting the SSD in the male‐biased direction in five *Belostoma* and one *Lethocerus* species. Our results also show that the family Belostomatidae has a relatively high proportion of male‐biased species. Moreover, the intensification of offspring care behaviour leads to a decrease in SSD in body length (Ohba et al. 2019) similar to that observed in *Eleutherodactylus* frogs (Dugo‐Cota et al. 2022). All these issues may explain the relatively low SDI values for species in littoral(o) habitats.

We acknowledge that there are difficulties in interpreting the effects of habitats on SSD. The habitat effect is not easy to interpret because SSD is the result of many different factors (abiotic factors, physical structure of the habitat, food sources, etc.). In the absence of quantitative measurements, we cannot say for certain which ecological factors influenced the phenotype of the sexes (Scordato et al. 2014). Furthermore, although we have an extensive database on the body length of the sexes, we have very little information on courtship, mating and sexual behaviour of most species. Another constraint is that our findings are based on a single trait, the body length. It is known, however, that sexual dimorphism can appear differently on several traits of insects (Blanckenhorn et al. 2007; Machado et al. 2021; Smith et al. 2024). To give just a few examples of the variety of possible characteristics, smaller males of water beetle (Dytiscinae) species with adhesive pads are favoured because they have to exert less force during mating than males of species without adhesive pads (Polido et al. 2024). In five *Belostoma* species, body length did not differ between sexes, but the middle and hind legs showed sexual dimorphism (Iglesias et al. 2010, 2012). Sex‐specific cuticular hydrocarbons may also act as sex‐specific traits, as has been shown in the field cricket *Teleogryllus oceanicus* (Thomas and Simmons 2008).

### 4.3 Are the Evolution of Allometry and Sexual Dimorphism Correlated?

According to the genetic theory, there is a positive correlation between the allometric slope of genus and SDI when selection is primarily directed to males (De Lisle and Rowe 2013). If the correlation is negative, we can assume selection is stronger in females (De Lisle and Rowe 2013). Like in copepods (Hirst and Kiørboe 2014) and in fishes (Horne et al. 2020), we did not find a significant correlation between allometric slope and SDI in the case of Nepomorpha. This conclusion suggests that the intensity of selection on females and males may be similar on the macroevolutionary level in this taxon. Furthermore, the fact that the variance in body length of females and males is not different in any of the genera we studied also supports the hypothesis that the intensity of selection on the sexes is comparable. If the selection of the two sexes is similar, then the SSD increases only if the variance of the body sizes is different (Cheverud et al. 1985). Since the variance in body length among Nepomopha is small, this may explain the relatively high number of monomorphic species.

### 4.4 Rensch's Rule Is a Weak Pattern in Aquatic Bugs

Nepomorpha exhibits ConRR, as observed by Teder and Tammaru (2005) in most female‐biased insect taxa. But for the infraorder Gerromorpha, which is taxonomically closest to the Nepomorpha, RR was found (Blanckenhorn et al. 2007). Moreover, Webb and Freckleton (2007) dispute the claim that RR is common in female‐biased taxa. We explain the reason for the ConRR in Nepomorpha following Dale et al. (2007), who divided the potential causes into three broad groups of causes: evolutionary constraint, natural selection and sexual selection. According to the evolutionary constraint, the pattern we observed may occur when the variance of genetic elements determining the body length of the two sexes is different and females respond more vigorously to selection pressure (Leutenegger and Cheverud 1982). There are no data on such evolutionary constraints in Nepomorpha. The effect of natural selection on SSD may be a consequence of competition between the sexes (Fairbairn 1997). According to currently available data, there is no competition between the sexes. So, it is unlikely that this factor would determine the occurrence of significant SDI. It is more likely that sexual selection (including fecundity selection; Alonzo and Servedio 2019) is widespread in Nepomorpha, as has been demonstrated in belostomatids (Tallamy 2001; Ohba 2019) and corixids (Nosil 2001; Candolin 2005; Sueur et al. 2011). If sexual selection has a stronger effect on females than on males, ConRR is to be expected (Dale et al. 2007) as we found in our study. At the superfamily, family and genus level, we did not see a clear pattern. We often found completely different allometries between the body lengths of the sexes in closely related taxa. This suggests that selection mechanisms need to be considered on a taxon‐by‐taxon basis, as exemplified, e.g., by belostomatids.

## 5 Conclusions

We know little about the evolution and development of sex differences in species of the Nepomorpha except for Belostomatidae. Based on the body length and its variance we have shown that more than 40% of species are monomorphic. Given the species' morphological diversity, it seems likely that other traits (including physiological, ecological and behavioural ones) besides body length must also be subjected to selection pressures. Monomorphism in Nepomorpha species has received little attention, although the question of whether monomorphism is an ancestral or a derived condition needs to be clarified. We hypothesise that different environmental factors in dissimilar habitats shape their SSD differently. However, detailed studies are needed to understand which particular environmental factors contribute to developing sex‐specific phenotypes and in what way. The present study found no evidence that phylogenetic relationships influence the validity of RR at the superfamily, family and genus levels. However, more detailed studies could clarify this issue more deeply in the future.

## Author Contributions

**Gábor Bakonyi:** conceptualization (lead), data curation (lead), formal analysis (lead), investigation (lead), methodology (equal), supervision (lead), validation (equal), writing – original draft (equal), writing – review and editing (equal). **Anikó Seres:** funding acquisition (equal), project administration (equal), resources (equal), writing – review and editing (equal).

## Conflicts of Interest

The authors declare no conflicts of interest.

## Supporting information


**Table S1.** Female and male body length data for 1195 species, subspecies and wing‐morph. The data and species names are presented as in the original articles.
**Table S2.** Those publications are presented in this table where the minimum and maximum body lengths of the species were given along with the averages. We calculated the arithmetic mean of the minimum and maximum data and compared it to the mean of the measurements on many individuals. Female and male data are presented in separate tables because not all publications included data for both sexes.
**Table S3.** Classification of aquatic bug genus into seven habitat types, with an indication of the publication from which we made the classification. We also present the genera belonging to Group 1 and Group 2.
**Table S4.** Body length data for the species for which we were able to perform a t‐test for statistical differences between sexes.
**Table S5.** Some important statistical parameters of the Sexual Dimorphism Index (SDI) of the 21 genera and a description of which genus is female‐biased, male‐biased and where there is no difference in the SDI value.
**Table S6.** Comparison of coefficients of variation for SDI values of species in different habitats (cvequality test). Upper triangle: D_AD values. Lower triangle: significance levels.

## Data Availability

The data that support the findings of this study are available in the Supporting Information of this article.

## References

[ece371501-bib-0001] Abouheif, E. , and D. J. Fairbairn . 1997. “A Comparative Analysis of Allometry for Sexual Size Dimorphism – Assessing Renschs Rule.” American Naturalist 149, no. 3: 540–562. 10.1086/286004.

[ece371501-bib-0002] Aiken, R. B. 1985. “Sound Production by Aquatic Insects.” Biological Reviews 60, no. 2: 163–211. 10.1111/j.1469-185X.1985.tb00714.x.

[ece371501-bib-0003] Alonzo, S. H. , and M. R. Servedio . 2019. “Grey Zones of Sexual Selection: Why Is Finding a Modern Definition So Hard?” Proceedings of the Royal Society B: Biological Sciences 286, no. 1909: 20191325. 10.1098/rspb.2019.1325. PMC6732385 31431160

[ece371501-bib-0004] Bailey, P. C. E. 1986. “The Feeding Behaviour of a Sit and Wait Predator Ranatra Dispar (Heteroptera: Nepidae): The Effect of Prey Density and Age Structure on the Number of Prey Eaten.” Ethology 72, no. 4: 277–286. 10.1111/j.1439-0310.1986.tb00629.x.

[ece371501-bib-0005] Bakonyi, G. 1978. “Contribution to the Knowledge of the Feeding Habits of Some Water Boatmen: Sigara spp. (Heteroptera: Corixidae).” Folia Entomologica Hungarica 31, no. 2: 19–24.

[ece371501-bib-0006] Bakonyi, G. 1984. “On the Morphology of the Palar Pegs of Some Corixidae (Heteroptera).” Acta Zoologica Hungarica 30, no. 3–4: 249–255.

[ece371501-bib-0007] Bakonyi, G. , E. Peták , T. Erős , and P. Sály . 2016. “Some Morphological Characteristics of the Water Scorpion Nepa Cinerea (Heteroptera: Nepomorpha) Are Associated With Habitat Type.” Acta Zoologica Academiae Scientiarum Hungaricae 62, no. 4: 369–385. 10.17109/AZH.62.4.369.2016.

[ece371501-bib-0008] Barske, J. , B. A. Schlinger , M. Wikelski , and L. Fusani . 2011. “Female Choice for Male Motor Skills.” Proceedings of the Royal Society B: Biological Sciences 278, no. 1724: 3523–3528. 10.1098/rspb.2011.0382. PMC3189371 21508030

[ece371501-bib-0009] Baur, J. , J. Roy , M. A. Schäfer , N. Puniamoorthy , W. U. Blanckenhorn , and P. T. Rohner . 2020. “Intraspecific Mating System Evolution and Its Effect on Complex Male Secondary Sexual Traits: Does Male–Male Competition Increase Selection on Size or Shape?” Journal of Evolutionary Biology 33, no. 3: 297–308. 10.1111/jeb.13565. 31701605

[ece371501-bib-0010] Bidau, C. J. , A. Taffarel , and E. R. Castillo . 2016. “Breaking the Rule: Multiple Patterns of Scaling of Sexual Size Dimorphism With Body Size in Orthopteroid Insects.” Revista de La Sociedad Entomologica Argentina 75, no. 2: 11–36.

[ece371501-bib-0011] Biró, A. , G. Balázs , Ž. Fišer , C. Fišer , G. Horváth , and G. Herczeg . 2024. “From Darkness to Twilight: Morphological Divergence Between Cave and Surface‐Subterranean Ecotone Niphargus Species.” Ecology and Evolution 14, no. 8: e70061. 10.1002/ece3.70061. 39108570 PMC11301267

[ece371501-bib-0012] Blanckenhorn, W. U. 2000. “The Evolution of Body Size: What Keeps Organisms Small?” Quarterly Review of Biology 75, no. 4: 385–407. 11125698 10.1086/393620

[ece371501-bib-0013] Blanckenhorn, W. U. 2005. “Behavioral Causes and Consequences of Sexual Size Dimorphism.” Ethology 111, no. 11: 977–1016. 10.1111/j.1439-0310.2005.01147.x.

[ece371501-bib-0014] Blanckenhorn, W. U. , A. F. G. Dixon , D. J. Fairbairn , et al. 2007. “Proximate Causes of Rensch's Rule: Does Sexual Size Dimorphism in Arthropods Result From Sex Differences in Development Time?” American Naturalist 169, no. 2: 245–257. 10.1086/510597. 17211807

[ece371501-bib-0015] Blanckenhorn, W. U. , R. Meier , and T. Teder . 2007. “Rensch's Rule in Insects: Patterns Among and Within Species.” In Sex, Size and Gender Roles: Evolutionary Studies of Sexual Size Dimorphism, edited by D. J. Fairbairn , W. U. Blanckenhorn , and T. Székely , 60–70. Oxford University Press.

[ece371501-bib-0016] Boisseau, R. P. , M. M. Ero , S. Makai , L. J. G. Bonneau , and D. J. Emlen . 2020. “Sexual Dimorphism Divergence Between Sister Species Is Associated With a Switch in Habitat Use and Mating System in Thorny Devil Stick Insects.” Behavioural Processes 181: 104263. 10.1016/j.beproc.2020.104263. 33049376

[ece371501-bib-0017] Bonduriansky, R. 2001. “The Evolution of Male Mate Choice in Insects: A Synthesis of Ideas and Evidence.” Biological Reviews of the Cambridge Philosophical Society 76, no. 3: 305–339. 10.1017/S1464793101005693. 11569787

[ece371501-bib-0018] Brown, L. N. , and J. E. McPherson . 1994. “Life History and Laboratory Rearing of Gelastocoris Oculatus Oculatus (Fabricius) (Hemiptera: Gelastocoridae) With Descriptions of Immature Stages.” Proceedings of the Entomological Society of Washington 96, no. 3: 516–526.

[ece371501-bib-0019] Butler, M. A. , T. W. Schoener , and J. B. Losos . 2000. “The Relationship Between Sexual Size Dimorphism and Habitat Use in Greater Antillean Anolis Lizards.” Evolution 54, no. 1: 259–272. 10.1111/j.0014-3820.2000.tb00026.x. 10937202

[ece371501-bib-0020] Byers, J. , E. Hebets , and J. Podos . 2010. “Female Mate Choice Based Upon Male Motor Performance.” Animal Behaviour 79, no. 4: 771–778. 10.1016/j.anbehav.2010.01.009.

[ece371501-bib-0021] Candolin, U. 2004. “Opposing Selection on a Sexually Dimorphic Trait Through Female Choice and Male Competition in a Water Boatman.” Evolution 58, no. 8: 1861–1864. 10.1111/j.0014-3820.2004.tb00470.x. 15446439

[ece371501-bib-0022] Candolin, U. 2005. “Why Do Multiple Traits Determine Mating Success? Differential Use in Female Choice and Male Competition in a Water Boatman.” Proceedings of the Royal Society B: Biological Sciences 272, no. 1558: 47–52. 10.1098/rspb.2004.2932. PMC1634947 15875569

[ece371501-bib-0023] Cheverud, J. M. , M. M. Dow , and W. Leutenegger . 1985. “The Quantitative Assessment of Phylogenetic Constraints.” Evolution 39, no. 6: 1335–1351. 28564267 10.1111/j.1558-5646.1985.tb05699.x

[ece371501-bib-0024] Chown, S. L. , and K. J. Gaston . 2010. “Body Size Variation in Insects: A Macroecological Perspective.” Biological Reviews 85, no. 1: 139–169. 10.1111/j.1469-185X.2009.00097.x. 20015316

[ece371501-bib-0025] Cloarec, A. 1988. “Behavioral Adaptations to Aquatic Life in Insects: An Example.” Advances in the Study of Behavior 18: 99–151. 10.1016/S0065-3454(08)60311-3.

[ece371501-bib-0026] Cloarec, A. 1996. “Variations of Behavioural Patterns During Development in the Water Stick Insect.” Journal of Ethology 14: 15–20.

[ece371501-bib-0027] Dale, J. , P. O. Dunn , J. Figuerola , T. Lislevand , T. Székely , and L. A. Whittingham . 2007. “Sexual Selection Explains Rensch's Rule of Allometry for Sexual Size Dimorphism.” Proceedings of the Royal Society B: Biological Sciences 274, no. 1628: 2971–2979. 10.1098/rspb.2007.1043. PMC2211517 17878139

[ece371501-bib-0028] Darwin, C. 1872. The Descent of Man and Selection in Relation to Sex. D. Appleton & Company.

[ece371501-bib-0029] De Lisle, S. P. , and L. Rowe . 2013. “Correlated Evolution of Allometry and Sexual Dimorphism Across Higher Taxa.” American Naturalist 182, no. 5: 630–639. 10.1086/673282. 24107370

[ece371501-bib-0030] Dugo‐Cota, A. , C. Vilà , A. Rodríguez , and A. Gonzalez‐Voyer . 2022. “Influence of Microhabitat, Fecundity, and Parental Care on the Evolution of Sexual Size Dimorphism in Caribbean Eleutherodactylus Frogs.” Evolution 76, no. 12: 3041–3053. 10.1111/evo.14642. 36210654 PMC10091758

[ece371501-bib-0031] Esperk, T. , T. Tammaru , S. Nylin , and T. Teder . 2007. “Achieving High Sexual Size Dimorphism in Insects: Females Add Instars.” Ecological Entomology 32, no. 3: 243–256. 10.1111/j.1365-2311.2007.00872.x.

[ece371501-bib-0032] Fairbairn, D. J. 1997. “Allometry for Sexual Size Dimorphism: Pattern and Process in the Coevolution of Body Size in Males and Females.” Annual Review of Ecology and Systematics 28: 659–687. 10.1146/annurev.ecolsys.28.1.659.

[ece371501-bib-0033] Faul, F. , E. Erdfelder , A.‐G. Lang , and A. Buchner . 2007. “GPower: A General Power Analysis Program.” Behavior Research Methods 39, no. 2: 175–191. 17695343 10.3758/bf03193146

[ece371501-bib-0034] García‐Navas, V. , T. Bonnet , R. Bonal , and E. Postma . 2016. “The Role of Fecundity and Sexual Selection in the Evolution of Size and Sexual Size Dimorphism in New World and Old World Voles (Rodentia: Arvicolinae).” Oikos 125, no. 9: 1250–1260. 10.1111/oik.03026.

[ece371501-bib-0035] García‐Navas, V. , V. Noguerales , P. J. Cordero , and J. Ortego . 2017. “Ecological Drivers of Body Size Evolution and Sexual Size Dimorphism in Short‐Horned Grasshoppers (Orthoptera: Acrididae).” Journal of Evolutionary Biology 30, no. 8: 1592–1608. 10.1111/jeb.13131. 28609564

[ece371501-bib-0036] Graça, M. B. , P. A. C. L. Pequeno , E. Franklin , and J. W. Morais . 2017. “Coevolution Between Flight Morphology, Vertical Stratification and Sexual Dimorphism: What Can we Learn From Tropical Butterflies?” Journal of Evolutionary Biology 30, no. 10: 1862–1871. 10.1111/jeb.13145. 28714129

[ece371501-bib-0037] Hädicke, C. W. , D. Rédei , and P. Kment . 2017. “The Diversity of Feeding Habits Recorded for Water Boatmen (Heteroptera: Corixoidea) World‐Wide With Implications for Evaluating Information on the Diet of Aquatic Insects.” European Journal of Entomology 114: 147–159. 10.14411/eje.2017.020.

[ece371501-bib-0038] Hammer, Ø. , D. A. T. Harper , and P. D. Ryan . 2001. “PAST: Paleontological Statistics Software Package for Education and Data Analysis.” Palaeontologia Electronica 4, no. 1: 9.

[ece371501-bib-0039] Heino, J. 2008. “Patterns of Functional Biodiversity and Function‐Environment Relationships in Lake Littoral Macroinvertebrates.” Limnology and Oceanography 53, no. 4: 1446–1455. 10.4319/lo.2008.53.4.1446.

[ece371501-bib-0040] Hirst, A. G. , and T. Kiørboe . 2014. “Macroevolutionary Patterns of Sexual Size Dimorphism in Copepods.” Proceedings of the Royal Society B: Biological Sciences 281, no. 1791: 20140739. 10.1098/rspb.2014.0739. PMC4132671 25100692

[ece371501-bib-0041] Horne, C. R. , A. G. Hirst , and D. Atkinson . 2020. “Selection for Increased Male Size Predicts Variation in Sexual Size Dimorphism Among Fish Species.” Proceedings of the Royal Society B: Biological Sciences 287, no. 1918: 20192640. 10.1098/rspb.2019.2640. PMC7003453 31937230

[ece371501-bib-0042] Hufnagel, L. , G. Bakonyi , and T. Vásárhelyi . 1999. “New Approach for Habitat Characterization Based on Species Lists of Aquatic and Semiaquatic Bugs.” Environmental Monitoring and Assessment 58, no. 3: 305–316. 10.1023/A:1006047130545.

[ece371501-bib-0043] Hungerford, H. B. 1948. “The Corixidae of the Western Hemisphere (Hemiptera).” University of Kansas Science Bulletin 32: 827.

[ece371501-bib-0044] Hyun, H. , B. Lee , and C. S. Han . 2024. “Repeated Copulation and Guarding, and Their Relationship With Male and Female Morphological Traits in the Water Scorpion Nepa Hoffmanni.” Ecology and Evolution 14, no. 12: e70725. 10.1002/ece3.70725. 39717631 PMC11664208

[ece371501-bib-0045] Ichikawa, N. 1989. “Repeated Copulations Benefit of the Female in Lethocerus Deyrollei Vuillefroy (Heteroptera: Belostomatidae).” Journal of Ethology 7, no. 2: 113–117. 10.1007/BF02350032.

[ece371501-bib-0046] Iglesias, M. S. , F. A. Crespo , and A. d. C. Valverde . 2012. “Is Parental Care Behavior in Belostoma Species an Evolutionary Cause for Their Common Sexual Size Dimorphism Pattern?” Entomological Science 15, no. 2: 155–161. 10.1111/j.1479-8298.2011.00499.x.

[ece371501-bib-0047] Iglesias, M. S. , A. C. Valverde , M. S. Gaspe , and F. A. Crespo . 2010. “Occurrence of Sexual Size Dimorphism in Belostoma Elegans (Heteroptera: Belostomatidae).” Entomological News 121, no. 1: 31–34. 10.3157/021.121.0106.

[ece371501-bib-0048] Keffer, S. L. , and J. E. McPherson . 1993. “Curictan Copulation and Waterscorpion Higher Classification (Heteroptera: Nepidae).” Proceedings of the Entomological Society of Washington 95, no. 1: 74–78.

[ece371501-bib-0049] Keith, D. , and E. McKevan . 1942. “Some Observations on Mononyx Nepaeformis (Fabricius 1775), a Toad‐Bug (Mononychidae, Hemipt.‐Heteropt.).” Proceedings of the Royal Entomological Society of London. Series A 17: 10–12.

[ece371501-bib-0050] Khandelwal, B. , and Y. C. Gupta . 2017. “Bionomics of Common Indian Water Scorpion, Laccotrephes Maculatus Fabr. (Hemiptera, Heteroptera: Nepidae).” Indian Research Journal of Genetics and Biotechnology 9, no. 2: 226–234.

[ece371501-bib-0051] Kojima, W. 2019. “Greater Degree of Body Size Plasticity in Males Than Females of the Rhinoceros Beetle Trypoxylus Dichotomus.” Applied Entomology and Zoology 54, no. 3: 239–246. 10.1007/s13355-019-00618-0.

[ece371501-bib-0052] Letunic, I. , and P. Bork . 2024. “Interactive Tree of Life (ITOL) v6: Recent Updates to the Phylogenetic Tree Display and Annotation Tool.” Nucleic Acids Research 52: W78–W82. 10.1093/nar/gkae268. 38613393 PMC11223838

[ece371501-bib-0053] Leutenegger, W. , and J. Cheverud . 1982. “Correlates of Sexual Dimorphism in Primates: Ecological and Size Variables.” International Journal of Primatology 3, no. 4: 387–402. 10.1007/BF02693740.

[ece371501-bib-0054] Li, M. , Y. Tian , Y. Zhao , and W. Bu . 2012. “Higher Level Phylogeny and the First Divergence Time Estimation of Heteroptera (Insecta: Hemiptera) Based on Multiple Genes.” PLoS One 7, no. 2: e32152. 10.1371/journal.pone.0032152. 22384163 PMC3288068

[ece371501-bib-0055] Liang, T. , S. Meiri , and L. Shi . 2022. “Sexual Size Dimorphism in Lizards: Rensch's Rule, Reproductive Mode, Clutch Size, and Line Fitting Method Effects.” Integrative Zoology 17, no. 5: 787–803. 10.1111/1749-4877.12569. 34216109

[ece371501-bib-0056] Lopes, C. F. , F. Stefanello , C. Bugs , C. Stenert , L. Maltchik , and J. R. I. Ribeiro . 2020. “Sexual Dimorphism in Belostoma Angustum Lauck (Insecta: Heteroptera: Belostomatidae) May be Related to Paternal Care.” Biological Journal of the Linnean Society 129, no. 2: 288–314. 10.1093/biolinnean/blz178.

[ece371501-bib-0057] Lovich, J. E. , and J. W. Gibbons . 1992. “A Review of Techniques for Quantifying Sexual Size Dimorphism.” Growth, Development, and Aging 56: 269–281. 1487365

[ece371501-bib-0058] Lynch, R. , H. Wasielewski , and L. Cronk . 2018. “Sexual Conflict and the Trivers‐Willard Hypothesis: Females Prefer Daughters and Males Prefer Sons.” Scientific Reports 8, no. 1: 15463. 10.1038/s41598-018-33650-1. 30337613 PMC6193998

[ece371501-bib-0059] Machado, G. , B. A. Buzatto , and D. S. M. Samia . 2021. “It Is Not Always About Body Size: Evidence of Rensch's Rule in a Male Weapon.” Biology Letters 17, no. 6: 20210234. 10.1098/rsbl.2021.0234. 34157237 PMC8219403

[ece371501-bib-0060] Maia‐Barbosa, P. M. , R. S. Peixoto , and A. S. Guimarães . 2008. “Zooplankton in Littoral Waters of a Tropical Lake: A Revisited Biodiversity.” Brazilian Journal of Biology 68, no. 4 Suppl: 1069–1078. 10.1590/S1519-69842008000500014. 19197477

[ece371501-bib-0061] Marwick, B. , and K. Krishnamoorthy . 2019. “Cvequality: Tests for the Equality of Coefficients of Variation From Multiple Groups.” R Software Package Version 0.1.3. https://github.com/benmarwick/cvequality.

[ece371501-bib-0062] Meiri, S. , and T. Liang . 2021. “Rensch's Rule—Definitions and Statistics.” Global Ecology and Biogeography 30, no. 3: 573–577. 10.1111/geb.13255.

[ece371501-bib-0063] Menke, A. S. 1979. “The Semiaquatic and Aquatic Hemiptera of California (Heteroptera: Hemiptera).” Bulletin of the California Insect Survey 21: 9568.

[ece371501-bib-0064] Nieser, N. 1967. “The Heteroptera of the Netherlands Antilles–VI. Notonectidae.” Studies on the Fauna of Curaçao and Other Caribbean Islands 96: 157–189.

[ece371501-bib-0065] Nieser, N. 1970. “Corixidae of Suriname and the Amazon With Records of Other Neotropical Species.” Studies on the Fauna of Suriname and Other Guyanas 54: 43–70.

[ece371501-bib-0066] Nieser, N. 1975. “The Water Bugs (Heteroptera: Nepomorpha) of the Guyana Region.” In Studies on the Fauna of Suriname and Other Guyanas, vol. 16, 1–308. Springer.

[ece371501-bib-0067] Nosil, P. 2001. “Sexual Size Dimorphism in a Natural Population of Callicorixa Vulnerata (Hemiptera: Corixidae).” Canadian Entomologist 133, no. 3: 311–313. 10.4039/Ent133311-3.

[ece371501-bib-0068] Ohba, S.‐y. 2019. “Ecology of Giant Water Bugs (Hemiptera: Heteroptera: Belostomatidae).” Entomological Science 22, no. 1: 6–20. 10.1111/ens.12334.

[ece371501-bib-0069] Ohba, S.‐y. , J. Ricardo , I. Ribeiro , and M. Santer . 2019. “Paternal Care in Giant Water Bugs.” In Aquatic Insects, edited by K. Del‐Claro and R. Guillermo , 321–340. Springer. 10.1007/978-3-030-16327-3_12.

[ece371501-bib-0070] Paetzold, A. , and K. Tockner . 2005. “Effects of Riparian Arthropod Predation on the Biomass and Abundance of Aquatic Insect Emergence.” Journal of the North American Benthological Society 24, no. 2: 395–402. 10.1899/04-049.1.

[ece371501-bib-0071] Pérez‐Barbería, F. J. , I. J. Gordon , and M. Pagel . 2002. “The Origins of Sexual Dimorphism in Body Size in Ungulates.” Evolution 56, no. 6: 1276–1285. 10.1111/j.0014-3820.2002.tb01438.x. 12144026

[ece371501-bib-0072] Pincheira‐Donoso, D. , and J. Hunt . 2017. “Fecundity Selection Theory: Concepts and Evidence.” Biological Reviews 92, no. 1: 341–356. 10.1111/brv.12232. 26526765

[ece371501-bib-0073] Polhemus, J. T. 2009. “Hemiptera (True Bugs).” In Encyclopedia of Inland Waters, 323–334. Elsevier.

[ece371501-bib-0074] Polhemus, J. T. 1994. “Stridulatory Mechanisms in Aquatic and Semiaquatic Heteroptera.” Journal of the New York Entomological Society 102, no. 2: 270–274.

[ece371501-bib-0075] Polhemus, J. T. , and D. A. Polhemus . 2008. “Global Diversity of True Bugs (Heteroptera; Insecta) in Freshwater.” Hydrobiologia 595, no. 1: 379–391. 10.1007/s10750-007-9033-1.

[ece371501-bib-0076] Polido, M. , V. M. Lopez , G. Gonzaga Da Silva , M. A. Del Lama , and R. Guillermo‐Ferreira . 2024. “A Diving Beetle's Dilemma: The Impact of Sexual Conflict on Rensch's Rule.” Biological Journal of the Linnean Society 142, no. 1: 38–43. 10.1093/biolinnean/blad120.

[ece371501-bib-0077] Popham, E. J. , T. M. Bryant , and A. A. Savage . 1984a. “The Function of the Abdominal Strigil in Male Corixid Bugs.” Journal of Natural History 18, no. 3: 441–444. 10.1080/00222938400770371.

[ece371501-bib-0078] Popham, E. J. , T. M. Bryant , and A. A. Savage . 1984b. “The Role of Front Legs of British Corixid Bugs in Feeding and Mating.” Journal of Natural History 18, no. 3: 445–464. 10.1080/00222938400770381.

[ece371501-bib-0079] Prenter, J. , W. I. Montgomery , and R. W. Elwood . 1997. “Sexual Dimorphism in Northern Temperate Spiders: Implications for the Differential Mortality Model.” Journal of Zoology 243, no. 2: 341–349. 10.1111/j.1469-7998.1997.tb02787.x.

[ece371501-bib-0080] Punzalan, D. , F. H. Rodd , and L. Rowe . 2008. “Contemporary Sexual Selection on Sexually Dimorphic Traits in the Ambush Bug Phymata Americana.” Behavioral Ecology 19, no. 4: 860–870. 10.1093/beheco/arn042.

[ece371501-bib-0081] Ramey, T. L. , and J. S. Richardson . 2017. “Terrestrial Invertebrates in the Riparian Zone: Mechanisms Underlying Their Unique Diversity.” Bioscience 67, no. 9: 808–819. 10.1093/biosci/bix078.

[ece371501-bib-0082] Rensch, B. 1950. “Die Abhängigkeit Der Relativen Sexualdifferenz von Der Körpergrösse.” Bonner Zoologische Beitraege 1: 58–69.

[ece371501-bib-0083] Rocha, P. N. , and F. M. Gawryszewski . 2024. “Foraging Strategy as a Route for Sexual Size Dimorphism Evolution.” Ecology and Evolution 14, no. 11: e70100. 10.1002/ece3.70100. 39512849 PMC11542996

[ece371501-bib-0084] Rohner, P. T. , T. Teder , T. Esperk , S. Lüpold , and W. U. Blanckenhorn . 2018. “The Evolution of Male‐Biased Sexual Size Dimorphism Is Associated With Increased Body Size Plasticity in Males.” Functional Ecology 32, no. 2: 581–591. 10.1111/1365-2435.13004.

[ece371501-bib-0085] Schoener, T. W. 1969. “Models of Optimal Size for Solitary Predators.” American Naturalist 103, no. 931: 277–313.

[ece371501-bib-0086] Schuh, R. T. , and J. A. Slater . 1995. True Bugs of the World (Hemiptera : Heteroptera) : Classification and Natural History. Cornell University Press.

[ece371501-bib-0087] Schuh, R. T. , and C. Weirauch . 2020. True Bugs of the World (Hemiptera: Heteroptera): Classification and Natural History. Siri Scientific Press.

[ece371501-bib-0088] Scordato, E. S. C. , L. B. Symes , T. C. Mendelson , and R. J. Safran . 2014. “The Role of Ecology in Speciation by Sexual Selection: A Systematic Empirical Review.” Journal of Heredity 105, no. S1: 782–794. 10.1093/jhered/esu037. 25149254

[ece371501-bib-0089] Shine, R. 1989. “Ecological Causes for the Evolution of Sexual Dimorphism: A Review of the Evidence.” Quarterly Review of Biology 64, no. 4: 419–461. 10.1086/416458. 2697022

[ece371501-bib-0090] Šigutová, H. , M. Šigut , A. Dolný , and F. Harabiš . 2022. “Individual Variability in Habitat Selection by Aquatic Insects Is Driven by Taxonomy Rather Than Specialisation.” Scientific Reports 12, no. 1: 20735. 10.1038/s41598-022-25363-3. 36456650 PMC9715563

[ece371501-bib-0091] Sites, R. W. 2022. “Phylogeny and Revised Classification of the Saucer Bugs (Hemiptera: Nepomorpha: Naucoridae).” Zoological Journal of the Linnean Society 195, no. 4: 1245–1286. 10.1093/zoolinnean/zlab105.

[ece371501-bib-0092] Sites, R. W. , and M. R. Willig . 2000. “Morphometric Variation Among Populations of Ambrysus Mormon Montadon (Heteroptera: Naucoridae).” Proceedings of the Entomological Society of Washington 102, no. 3: 533–541.

[ece371501-bib-0093] Slatkin, M. 1984. “Ecological Causes of Sexual Dimorphism.” Evolution 38, no. 3: 622–630. 28555984 10.1111/j.1558-5646.1984.tb00327.x

[ece371501-bib-0094] Slimani, N. , E. Guilbert , J. C. White , et al. 2022. “Unravelling the Environmental Correlates Influencing the Seasonal Biodiversity of Aquatic Heteropteran Assemblages in Northern Africa.” Limnologica 97: 126021. 10.1016/j.limno.2022.126021.

[ece371501-bib-0095] Smith, J. M. L. , A. M. Catherall‐Ostler , R. Mashoodh , and R. M. Kilner . 2024. “Sexual Dimorphism in Head Size in Wild Burying Beetles.” Ecological Entomology 49, no. 6: 846–856. 10.1111/een.13359.

[ece371501-bib-0096] Smith, R. L. 1979. “Repeated Copulation and Sperm Precedence: Paternity Assurance for a Male Brooding Water Bug.” Science 205: 1029–1031. 10.1126/science.205.4410.1029. 17795564

[ece371501-bib-0097] Stillwell, C. , W. U. Blanckenhorn , T. Teder , G. Davidowitz , and C. W. Fox . 2010. “Sex Differences in Phenotypic Plasticity Affect Variation in Sexual Size Dimorphism in Insects: From Physiology to Evolution.” Annual Review of Entomology 55: 227–245. 10.1146/annurev-ento-112408-085500. PMC4760685 19728836

[ece371501-bib-0098] Stonedahl, G. M. , and J. D. Lattin . 1986. The Corixidae of Oregon and Washington (Hemiptera: Heteroptera). Technical Bulletin, 150. Oregon Experiment Station, Oregon State University.

[ece371501-bib-0099] Strayer, D. L. , and S. E. G. Findlay . 2010. “Ecology of Freshwater Shore Zones.” Aquatic Sciences 72, no. 2: 127–163. 10.1007/s00027-010-0128-9.

[ece371501-bib-0100] Sueur, J. , D. Mackie , and J. F. C. Windmill . 2011. “So Small, So Loud: Extremely High Sound Pressure Level From a Pygmy Aquatic Insect (Corixidae, Micronectinae).” PLoS One 6, no. 6: e21089. 10.1371/journal.pone.0021089. 21698252 PMC3115974

[ece371501-bib-0101] Svensson, B. G. , B. Tallmark , and E. Petersson . 2000. “Habitat Heterogeneity, Coexistence and Habitat Utilization in Five Backswimmer Species (Notonecta Spp.; Hemiptera, Notonectidae).” Aquatic Insects 22, no. 2: 81–98. 10.1076/0165-0424(200004)22:2;1-P;FT081.

[ece371501-bib-0102] Tallamy, D. W. 2001. “Evolution of Exclusive Paternal Care in Arthopods.” Annual Review of Entomology 46, no. 1: 139–165. 10.1146/annurev.ento.46.1.139. 11112166

[ece371501-bib-0103] Tawfik, M. F. S. , and K. T. Awadallah . 1975. “The Biology of the Water‐Scorpion Ranatra Vicina Signoret (Heteroptera, Nepidae).” Deutsche Entomologische Zeitschrift NF 22: 229–240.

[ece371501-bib-0104] Teder, T. , A. Kaasik , K. Taits , and T. Tammaru . 2021. “Why Do Males Emerge Before Females? Sexual Size Dimorphism Drives Sexual Bimaturism in Insects.” Biological Reviews 96, no. 6: 2461–2475. 10.1111/brv.12762. 34128582

[ece371501-bib-0105] Teder, T. , and T. Tammaru . 2005. “Sexual Size Dimorphism Within Species Increases With Body Size in Insects.” Oikos 108, no. 2: 321–334. 10.1111/j.0030-1299.2005.13609.x.

[ece371501-bib-0106] Thomas, M. L. , and L. W. Simmons . 2008. “Sexual Dimorphism in Cuticular Hydrocarbons of the Australian Field Cricket Teleogryllus Oceanicus (Orthoptera: Gryllidae).” Journal of Insect Physiology 54, no. 6: 1081–1089. 10.1016/j.jinsphys.2008.04.012. 18519139

[ece371501-bib-0107] Trivers, R. 1972. “Parental Investment and Sexual Selection.” In Sexual Selection and the Descent of Man 1871–1971, edited by B. G. Campbell , 136–179. Aldine Pub. Co.

[ece371501-bib-0108] Truxal, F. S. 1949. “A Study of the Genus Martarega (Hemiptera, Notonectidae).” Journal of the Kansas Entomological Society 22, no. 1: 1–24.

[ece371501-bib-0109] Waitzbauer, W. 1976. “Energieumsatz Aquatischer Hemipteren.” Oecologia 22, no. 2: 179–209. 10.1007/bf00344715. 28308654

[ece371501-bib-0110] Walseng, B. , D. O. Hessen , G. Halvorsen , and A. K. Schartau . 2006. “Major Contribution From Littoral Crustaceans to Zooplankton Species Richness in Lakes.” Limnology and Oceanography 51, no. 6: 2600–2606. 10.4319/lo.2006.51.6.2600.

[ece371501-bib-0111] Wang, Y. , F. F. F. Moreira , D. Rédei , et al. 2020. “Diversification of True Water Bugs Revealed by Transcriptome‐Based Phylogenomics.” Systematic Entomology 46, no. 2: 339–356. 10.1111/syen.12465.

[ece371501-bib-0112] Webb, T. J. , and R. P. Freckleton . 2007. “Only Half Right: Species With Female‐Biased Sexual Size Dimorphism Consistently Break Rensch's Rule.” PLoS One 2, no. 9: e897. 10.1371/journal.pone.0000897. 17878932 PMC1964802

[ece371501-bib-0113] Weirauch, C. , R. T. Schuh , G. Cassis , and W. C. Wheeler . 2019. “Revisiting Habitat and Lifestyle Transitions in Heteroptera (Insecta: Hemiptera): Insights From a Combined Morphological and Molecular Phylogeny.” Cladistics 35, no. 1: 67–105. 10.1111/cla.12233. 34622978

[ece371501-bib-0114] Winkler, L. , R. P. Freckleton , T. Székely , and T. Janicke . 2024. “Pre‐Copulatory Sexual Selection Predicts Sexual Size Dimorphism: A Meta‐Analysis of Comparative Studies.” Ecology Letters 27, no. 9: e14515. 10.1111/ele.14515. 39354897

[ece371501-bib-0115] Wu, H. , T. Jiang , X. Huang , and J. Feng . 2018. “Patterns of Sexual Size Dimorphism in Horseshoe Bats: Testing Rensch's Rule and Potential Causes.” Scientific Reports 8: 2616. 10.1038/s41598-018-21077-7. 29422495 PMC5805768

[ece371501-bib-0116] Ye, Z. , J. Damgaard , C. W. Hädicke , et al. 2023. “Phylogeny and Historical Biogeography of the Water Boatmen (Insecta: Hemiptera: Heteroptera: Nepomorpha: Corixoidea).” Molecular Phylogenetics and Evolution 180: 107698. 10.1016/j.ympev.2022.107698. 36587885

[ece371501-bib-0117] Ye, Z. , J. Damgaard , H. Yang , M. B. Hebsgaard , T. Weir , and W. Bu . 2020. “Phylogeny and Diversification of the True Water Bugs (Insecta: Hemiptera: Heteroptera: Nepomorpha).” Cladistics 36, no. 1: 72–87. 10.1111/cla.12383. 34618947

[ece371501-bib-0118] Young, E. C. 2010. “The Taxonomic Impediment of Unrecognised Flight Polimorphysm in Notonectidae (Hemiptera: Heteroptera).” Zootaxa 2535: 35–48.

[ece371501-bib-0119] Zohary, T. , and A. Gasith . 2014. “The Littoral Zone.” In Lake Kinneret: Ecology and Management, edited by T. Zohary , A. Sukenik , T. Berman , and A. Nishri , 517–532. Springer Dordrecht.

